# Circulating Extracellular Vesicle Levels in Patients with Coronavirus Disease 2019 Coagulopathy: A Prospective Cohort Study

**DOI:** 10.3390/jcm12103460

**Published:** 2023-05-14

**Authors:** Yudai Iwasaki, Yusuke Takei, Mitsuhiro Yamada, Shigekazu Sugino, Koji Saito, Tetsuji Aoyagi, Kengo Oshima, Hajime Kanamori, Hiroaki Baba, Kentarou Takei, Koichi Tokuda, Eichi N. Kodama, Tetsuro Kamo, Tadashi Kamio, Takehiko Kasai, Satoru Ogawa, Masanori Yamauchi

**Affiliations:** 1 Department of Anesthesiology and Perioperative Medicine, Tohoku University Graduate School of Medicine, 2-1 Seiryo-machi, Aoba-ku, Sendai 980-8575, Miyagi, Japan; 2 Department of Respiratory Medicine, Tohoku University Graduate School of Medicine, 2-1 Seiryo-machi, Aoba-ku, Sendai 980-8575, Miyagi, Japan; 3 Department of Infectious Diseases, Internal Medicine, Tohoku University Graduate School of Medicine, 2-1 Seiryo-machi, Aoba-ku, Sendai 980-8575, Miyagi, Japan; 4 Division of Infectious Diseases, International Research Institute of Disaster Science, Graduate School of Medicine, Tohoku Medical Megabank Organization, Tohoku University, 2-1 Seiryo-machi, Aoba-ku, Sendai 980-8575, Miyagi, Japan; 5 Department of Emergency Medicine and Critical Care Medicine, Tochigi Prefectural Emergency and Critical Care Center, Imperial Gift Foundation Saiseikai, Utsunomiya Hospital, Utsunomiya-shi 321-0974, Tochigi, Japan; 6 Department of Intensive Care, Shonan Kamakura General Hospital, 1370-1 Okamoto, Kamakura 247-8533, Kanagawa, Japan; 7 Department of Emergency Medicine, Sapporo Medical University, South 1 West 17, Sapporo 060-8556, Hokkaido, Japan; 8 Department of Pain Management and Palliative Care Medicine, Kyoto Prefectural University of Medicine, 465 Kajii-cho, Kamigyo-ku, Kyoto 602-8566, Kyoto, Japan

**Keywords:** coagulopathy, COVID-19, extracellular vesicle, mechanism, prospective, observational

## Abstract

Coronavirus disease 2019 (COVID-19) is associated with coagulopathy. However, the underlying mechanisms are not completely understood. We evaluated the association between COVID-19 coagulopathy and extracellular vesicle (EV) levels. We hypothesized that several EV levels would be higher in COVID-19 coagulopathy patients than in non-coagulopathy patients. This prospective observational study was conducted in four tertiary care faculties in Japan. We enrolled 99 COVID-19 patients (48 with coagulopathy and 51 without coagulopathy) aged ≥20 years who required hospitalization, and 10 healthy volunteers; we divided the patients into coagulopathy and non-coagulopathy groups according to the D-dimer levels (≥1 μg/mL and <1 μg/mL, respectively). We used flow cytometry to measure the tissue-factor-bearing, endothelium-derived, platelet-derived, monocyte-derived, and neutrophil-derived EV levels in platelet-free plasma. The EV levels were compared between the two COVID-19 groups as well as among the coagulopathy patients, non-coagulopathy patients, and healthy volunteers. No significant difference was found in EV levels between the two groups. Meanwhile, the cluster of differentiation (CD) 41 + EV levels were significantly higher in COVID-19 coagulopathy patients than in healthy volunteers (549.90 [255.05–984.65] vs. 184.3 [150.1–254.1] counts/µL, *p* = 0.011). Therefore, CD41+ EVs might play an essential role in COVID-19 coagulopathy development.

## 1. Introduction

Coronavirus disease 2019 (COVID-19) is characterized by coagulopathy, with thrombosis occurring in approximately 30% of patients with severe COVID-19 requiring intensive care [1,2]. Pulmonary microembolism has also been observed in patients with COVID-19, including clinically asymptomatic patients, as identified using perfusion computed tomography or other imaging techniques [3].

Despite several hypotheses, such as vascular endothelial inflammation [4], increased mitogen-activated protein kinase signaling and thromboxane A2 production [5], the origin of this coagulopathy remains unclear. Some previous studies have reported an association between extracellular vesicles (EVs) and COVID-19 coagulopathy. EVs are small (0.05–1 μm), heterogeneous, cell-membrane-derived molecules released in response to specific stimuli, including apoptosis [6]. EVs include several intercellular communication molecules, such as plasma membrane proteins, messenger ribonucleic acid (RNA), and microRNAs [7]. The origins of EVs can be traced back to the cell-specific antigens, which can be used as cytotoxicity markers. The proinflammatory and hypercoagulable properties of EVs are well known, with endothelium- and platelet-derived EVs implicated in hypercoagulation [8,9,10,11]. Additionally, tissue factor (TF)-bearing EVs may initiate the coagulation cascade [12,13], and EVs contribute to the pathology of disseminated intravascular coagulation [14]. However, the association between EVs and COVID-19 coagulopathy has not yet been clarified.

Therefore, this study aimed to evaluate the relationship between COVID-19 coagulopathy and EV levels. We hypothesized that EV levels would be higher in COVID-19 coagulopathy patients than in non-coagulopathy patients. Due to the lack of studies examining specific EVs, we conducted exploratory research on several EVs that could be associated with coagulopathy. Flow cytometry was also insufficient at differentiating microvesicles from other exosomes or apoptotic bodies. Therefore, EVs were defined as vesicles of 0.1–1 μm in size that stain positive for Annexin V and with surfactant-disappearing properties in this study.

## 2. Materials and Methods

### 2.1. Study Design and Setting

This prospective, multicenter, observational study was conducted in the following four tertiary care hospitals in Japan: Tohoku University Hospital, Saiseikai Utsunomiya Hospital, Sapporo Medical University Hospital, and Shonan Kamakura General Hospital. This study was approved by the Institutional Review Board and Ethics Committee of the Tohoku University School of Medicine (ID 2020-1-660, approved on 28 October 2020). This study was registered with the University Hospital Medical Information Network (registration no. UMIN000018111) and adhered to the principles of the Declaration of Helsinki. Written informed consent was obtained from each volunteer and each patient or family member prior to study inclusion.

### 2.2. Healthy Volunteer Group and Patient Selection

#### 2.2.1. Healthy Volunteers

Healthy volunteers were recruited from Tohoku University Hospital. Five healthy men and five healthy women aged 20 years and older with no chronic diseases requiring regular medication, with a body mass index of <30 kg/m^2^, with no history of smoking, with no history of COVID-19, and who were not pregnant during the study comprised the healthy volunteer group.

#### 2.2.2. Patients

Patients aged ≥20 years who were diagnosed with COVID-19 and required hospitalization were included in this study. The diagnosis was made using a polymerase chain reaction, antigen test, or loop-mediated isothermal amplification [15,16,17]. Meanwhile, patients who did not provide consent or whose close relatives did not provide consent; patients with other infections in addition to COVID-19; patients with end-stage heart failure defined as class IV according to the New York Heart Association Functional Classification [18], end-stage liver failure defined as Child-Pugh classification C [19], and any malignancy undergoing treatment; pregnant women; patients on their second admission for COVID-19; and patients accidentally diagnosed with COVID-19 through investigations of diseases requiring emergency therapeutic interventions were excluded.

#### 2.2.3. Group Assignment

The enrolled COVID-19 patients were divided into the non-coagulopathy group (D-dimer level <1 μg/mL) and coagulopathy group (D-dimer level ≥1 μg/mL) [20].

### 2.3. Data Collection

We extracted the following baseline characteristics from the medical records of the enrolled patients: age; sex; height; weight; body mass index; smoking history; comorbidities before admission according to the Charlson Comorbidity Index [21]; history of venous thromboembolism; anticoagulant or antiplatelet therapy before hospitalization; blood cell counts; lactate dehydrogenase level; total bilirubin level; total protein level; total albumin level; blood urea nitrogen level; creatinine level; C-reactive protein level; activated partial thromboplastin time; fibrinogen level; D-dimer level; clinical progression status of COVID-19 [22]; therapeutic interventions provided, such as oxygen administration, mechanical ventilation, and venovenous extracorporeal membrane oxygenation; length of hospital stay; length of intensive care unit stay; and discharge outcome. The discharge outcome was categorized as home discharge, transfer to another hospital, or death. No information regarding the healthy volunteer group was collected.

### 2.4. Outcomes

We postulated that COVID-19 coagulopathy would be associated with an increase in EV levels. However, before this study, no evidence was available on the association between EV levels and COVID-19 coagulopathy. Therefore, we conducted a preliminary study to confirm our hypothesis by comparing several EV levels between a healthy volunteer group and the first 10 patients from the coagulopathy group. Next, we compared the EV levels between the COVID-19 coagulopathy and non-coagulopathy groups. Due to the exploratory nature of our research and the need to detect EVs linked to coagulopathy, we examined the levels of multiple EV types.

The primary endpoint was the difference in levels of TF-bearing EVs between the coagulopathy and non-coagulopathy groups, while the secondary outcomes were the levels of other types of EVs. In addition to TF-bearing EVs, we measured the levels of endothelium-derived EVs, platelet-derived EVs, monocyte-derived EVs, and neutrophil-derived EVs as the target EVs. We also measured the levels of each EV combined with TF.

### 2.5. Experiment Methodology for EVs Analysis

#### 2.5.1. Sample Collection and Treatment

Blood samples were collected from patients with COVID-19 within 72 hours after admission. After collection of whole blood in citrate acid-coated evacuated tubes (VP-CA050K70; TERUMO, Tokyo, Japan), the samples were centrifuged at 2500× *g* for 15 min, and the isolated supernatant was centrifuged at 2500× *g* for 15 min to prepare the platelet-free plasma (PFP) according to the methodological guidelines [23,24]. The PFP was stored at −80 °C until analysis.

#### 2.5.2. Detection of EVs and Definition of Target EVs

**Figure 1:**
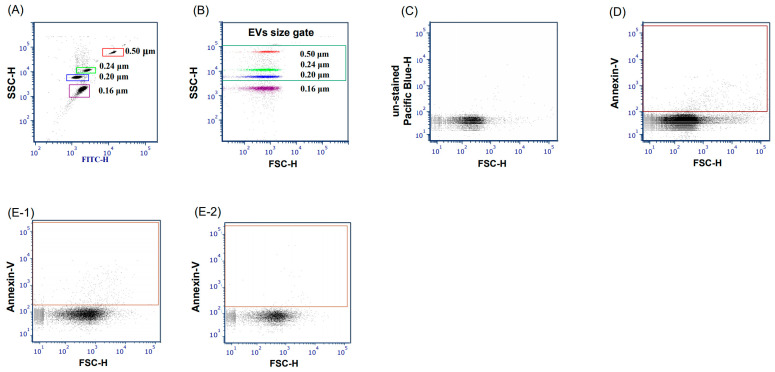
Flow cytometric analysis and determination of each gate to detect the target EVs. (**A**) A region was determined around each singlet bead population using a mix of fluorescent beads of various diameters (Megamix Plus SSC™). (**B**) The EV size gate was defined as a green-lined rectangle according to the distribution of bead singles at 0.20 (blue), 0.24 (light green), and 0.5 μm (red). This EV size gate covers the theoretical medium EV size range. (**C**) Non-stain human plasma was utilized as the negative control for the Annexin Ⅴ stain. (**D**) EVs were defined as 0.1–1.0 μm sized and Annexin V-positive populations. The EV gate was set as a brown-lined rectangle. (**E**) Flow cytometry analysis of human plasma before treatment with Triton-X (**E-1**) and after treatment with Triton-X (**E-2**). EVs were not detected after treatment with Triton-X, indicating that visible small vesicles were derived from the extracellular membrane.

For the counting and characterization of EVs in PFP, flow cytometry (BD LSRFortessa; BD Biosciences, San Jose, CA, USA) was used. EVs are small vesicles derived from the plasma membrane, and are conventionally referred to as microparticles. Therefore, we defined EVs as phosphatidylserine-positive particles of <1 μm in size. To set the region for subpopulations of particles measuring 0.1–1.0 μm in size, Megamix-Plus SSC fluorescent polystyrene beads (Biocytex, Marseille, France) were used. Additionally, we used Brilliant Violet 421 (BV421)-conjugated Annexin V to detect phosphatidylserine-positive particles (Figure 1). To count the absolute EV number, a known concentration of counting spheres (1000 spheres/μL; Flow-Count Fluorospheres; Beckman Coulter, Brea, CA, USA) was added to each sample as an internal standard.

We measured the levels of TF-bearing EVs (CD142^+^ EVs), endothelium-derived EVs (CD31^+^/CD41^−^ EVs or CD62E^+^ EVs), platelet-derived EVs (CD41^+^ EVs or CD62P^+^ EVs), neutrophil-derived EVs (CD16b^+^ EVs), and monocyte-derived EVs (CD14^+^ EVs). As the platelet endothelial cell adhesion molecule (PECAM: CD31) is expressed in platelets and endothelial cells, we defined endothelium-derived EVs as CD31^+^/CD41^−^ EVs to differentiate them from platelet-derived CD31^+^ EVs using CD41, a commonly used platelet marker.

Overall, 10 μL of freshly thawed PFP was incubated with fluorescent conjugated monoclonal antibodies against each cell-specific antigen for 30 min in the dark at room temperature. Subsequently, the samples were diluted with 100 μL of Annexin V binding buffer (Biolegend, San Diego, USA) and incubated with BV421-conjugated Annexin V (Biolegend) for 15 min in the dark at room temperature. After incubation, the samples were diluted with 1000 μL of Annexin V binding buffer, followed by centrifugation at 20,000× *g* for 45 min to obtain an EV-rich pellet. At our institution, viral inactivation treatment of the samples was performed in experiments that involved the use of samples from COVID-19 patients in a collaborative laboratory. Therefore, after removing 900 μL of the supernatant, we added 200 μL of Annexin V binding buffer containing paraformaldehyde and adjusted the final paraformaldehyde concentration to 2% to inactivate the severe acute respiratory syndrome coronavirus 2 (SARS-CoV-2) in the samples. To quantify the absolute number of EVs, 100 μL of the diluted samples was added to an equal volume of Flow-Count fluorosphere solution (Beckman Coulter). The samples were examined using flow cytometry for 2 min.

The following fluorescent conjugated monoclonal antibodies were used for the analysis of target EVs: allophycocyanin (APC)-conjugated anti-CD142 antibody (BioLegend; for TF^+^ EVs), fluorescent isothiocyanate (FITC)-conjugated anti-CD31 antibody (BD Pharmingen), and phycoerythrin (PE)-conjugated anti-CD41 antibody (Beckman Coulter) for CD31^+^CD41^−^ EVs; PE-conjugated CD62E antibody (BD Pharmingen) for CD62E^+^ EVs; PE-conjugated anti-CD41 antibody (Beckman Coulter) for CD41^+^ EVs; FITC-conjugated P-selectin (CD62P) antibody (Biolegend) for CD62P^+^ EVs; PE-conjugated CD14 antibody (Biolegend) for CD14^+^ EVs; and PE-conjugated CD16b antibody (BD Pharnigen) for CD16b^+^ EVs. The isotype controls against each antibody were used as negative controls.

In our protocol, four combinations of multiple immunostainings were performed to measure each EV combined with TF. The first combination was FITC-conjugated anti-CD31 antibody, PE-conjugated anti-CD41 antibody, and APC-conjugated anti-CD142 antibody. The second combination was FITC-conjugated CD62P antibody, PE-conjugated CD16b antibody, and APC-conjugated anti-CD142 antibody. The third combination was PE-conjugated CD62E antibody and APC-conjugated anti-CD142 antibody. The fourth combination was PE-conjugated CD14 antibody and APC-conjugated anti-CD142 antibody. In this immunostaining method, we measured the levels of TF-bearing EVs four times per sample. Subsequently, we calculated the mean value of TF-bearing EVs in each count to compare the levels of TF-bearing EVs.

#### 2.5.3. Detergent Analysis

To confirm whether the EVs detected using flow cytometry were derived from lipid-membrane-bound vesicles rather than from debris, detergent lysis was performed using Triton X-100 [25]. We added Triton X-100 to EV-rich fluids to adjust the final Triton X-100 concentration to 5%, and incubated the mixture at 4 °C overnight. We conducted all procedures in a Biosafety Level 3 laboratory.

### 2.6. Statistical Analysis

Continuous data are presented as means and standard deviations or medians and interquartile ranges, depending on the normality of the distribution. Categorical or binary variables are presented as counts and percentages.

A t-test was used to analyze continuous data with a normal distribution, while the Mann–Whitney U test was used to analyze continuous data with a non-normal distribution. Categorical variables were assessed using Fisher’s exact test. The patients’ baseline data were compared between the two groups using these tests. Subsequently, three comparisons were made using the data related to the type of EV, as follows: (1) a comparison between the first 10 enrolled patients with COVID-19 coagulopathy and 10 healthy volunteers to confirm the hypothesis as a preliminary study; (2) a comparison between the coagulopathy and non-coagulopathy groups; and (3) a comparison among all three groups (healthy volunteer, COVID-19 coagulopathy, and COVID-19 non-coagulopathy groups). For the analysis of comparisons 1 and 2, a t-test or Mann–Whitney U test was used based on the distribution of EV counts. For the analysis between the healthy volunteer and COVID-19 coagulopathy groups, the data of the first 10 enrolled patients were used. The Kruskal–Wallis test was used to analyze comparison 3, while Bonferroni’s correction was used to compare two groups, with the significance level set at a p-value of 0.017. For this analysis, we selected EVs that were statistically different between the COVID-19 coagulopathy and healthy volunteer groups in comparison 1. A two-sided p-value of  <0.05 was considered significant. All analyses were conducted using R version 4.1.1 software (10 August 2021) and GraphPad PRISM 6.0 (GraphPad Software Inc, California, United States).

We hypothesized that the EV levels would be significantly higher in the COVID-19 coagulopathy group than in the healthy volunteer group, and moderately higher in the COVID-19 coagulopathy group than in the COVID-19 non-coagulopathy group. When the sample size was calculated by comparing the healthy volunteer and COVID-19 coagulopathy groups, the following parameters were set: effect size of 1.4, α error of 0.05, power of 0.8, group ratio of 1:1. The Wilcoxon–Mann–Whitney test was set as a two-tailed test. The sample size was calculated to be 10 patients in each group. Based on this calculation, we recruited 10 healthy volunteers.

When we calculated the sample size by comparing the coagulopathy and non-coagulopathy groups, the following parameters were set: effect size of 0.8, α error of 0.05, power of 0.8, group ratio of 1:4 (because of the low reported proportion of COVID-19 coagulopathy [1,2]). The Wilcoxon–Mann–Whitney test was set as a two-tailed test. Based on this calculation and assuming a 10% proportion of missing data, we determined the sample size to be 100. G*power 3.1.9.7 software was used to calculate the sample size [26].

## 3. Results

### 3.1. Flow Chart of the Patient Selection Process and Patients’ Baseline Characteristics

**Figure 2:**
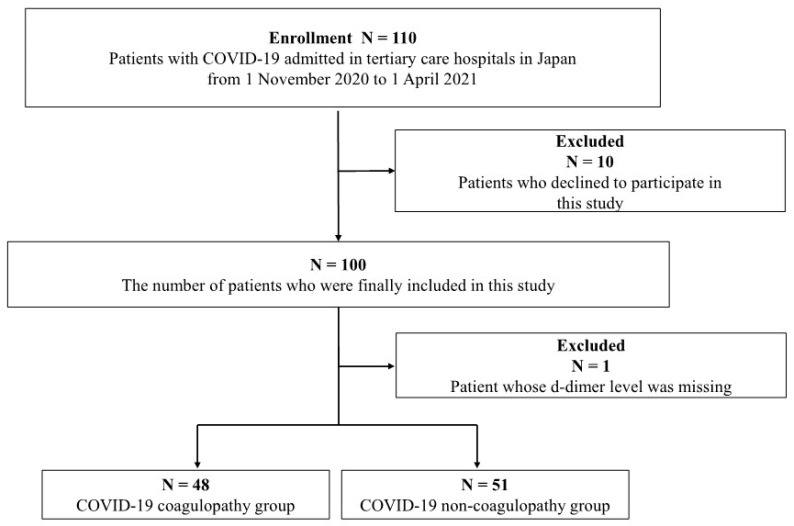
Flowchart of patient selection.

From November 2020 to April 2021, 110 COVID-19 patients were enrolled. Of these, 10 patients declined to participate; thus, 100 patients were initially included. As the information on the D-dimer level of 1 patient was missing, 99 were included in the final analysis. This cohort was divided into two groups based on the D-dimer levels. In total, 51 and 48 patients were enrolled in the non-coagulopathy and coagulopathy groups, respectively (Figure 2). In the same study period, 10 healthy controls were evaluated for EV detection.

Table 1 illustrates the coagulopathy and non-coagulopathy groups’ backgrounds and clinical outcomes during hospitalization. Age, dementia at admission, Charlson Comorbidity Index, and several laboratory findings, such as white blood cell counts, lactate dehydrogenase level, total protein level, albumin level, blood urea nitrogen level, C-reactive protein level, and fibrinogen level differed between the two groups. The coagulopathy group required oxygenation therapy at admission; this finding indicated that the disease severity in this group was higher than that in the non-coagulopathy group. Moreover, the coagulopathy group had worse clinical progression and a higher mortality rate than the non-coagulopathy group.

### 3.2. EV Comparison

The staining strategy used for the flow cytometric analysis of EVs is shown in Supplementary Material. Our methodology enabled the determination of all defined EVs. Triton-X treatment eliminated the Annexin V-positive population during the flow cytometric analysis, confirming that the small vesicles we measured and defined as EVs were derived from the cell plasma membrane (Figure 1E).

**Figure 3:**
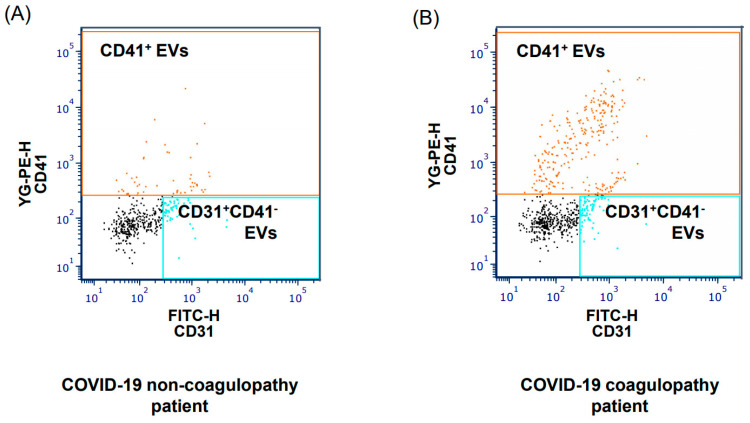
Representative plots of flow cytometric analysis for CD41+ EVs in patients with COVID-19 with (**A**) or without (**B**) coagulopathy. COVID-19, coronavirus disease 2019; EVs, extracellular vesicles.

Table 2 shows the comparison of EVs between the healthy volunteer group and 10 COVID-19 coagulopathy patients. The numbers of CD41^+^ EVs, CD14^+^ EVs, and CD142^+^ EVs were statistically higher in the COVID-19 coagulopathy group, thereby supporting our hypothesis of an association between COVID-19 coagulopathy and EVs. Table 3 summarizes the comparison of EVs between the non-coagulopathy and coagulopathy groups. No difference was found in the EV levels between the two groups, despite the higher proportion of patients in the COVID-19 coagulopathy group who had higher CD41+ EV levels than those in the COVID-19 non-coagulopathy group (Figure 3).

**Figure 4:**
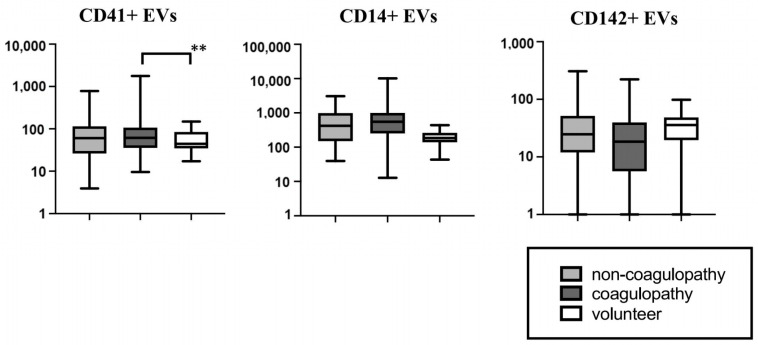
Comparison of extracellular vesicles among three groups. CD41+ EV levels were higher in patients with COVID-19 and coagulopathy among the three groups. COVID-19, coronavirus disease 2019; EVs, extracellular vesicles. “**” indicates that the *p*-value is less than 0.017, demonstrating a statistically significant difference.

Figure 4 illustrates the EV comparisons among the three groups. The analysis focused on the CD41^+^, CD14^+^, and CD142^+^ EVs, which were statistically different between the COVID-19 coagulopathy and the healthy volunteer groups. In the Kruskal–Wallis test, the CD41^+^ EV levels were statistically different among the groups (*p* = 0.027). Following Bonferroni correction, the coagulopathy group had higher CD41^+^ EV levels than the healthy volunteer group (549.90 [255.05–984.65] counts/µL vs. 184.3 [150.1–254.1] counts/µL, *p* = 0.011).

## 4. Discussion

In our study, COVID-19 patients with high D-dimer levels showed elevated levels of platelet-derived EVs, monocyte-derived EVs, and TF^+^ EVs compared with the healthy volunteer group; however, no difference was observed in the types of EVs between the coagulopathy and non-coagulopathy groups. In a previous study comparing the three groups, the CD41^+^ EV levels were significantly higher in the coagulopathy group than in the healthy volunteer group. This is the first study to compare the levels of several EVs among COVID-19 coagulopathy patients, COVID-19 non-coagulopathy patients, and healthy volunteers.

Previous studies demonstrated the elevation of EV levels in COVID-19 patients compared with that in healthy volunteers [27,28], and our research revealed similar results. Platelet-derived EVs interact with several types of blood cells, causing inflammatory reactions and coagulant stimulation [10,29]. These EVs regulate hemostasis in trauma patients with massive hemorrhage [30], which might cause hypercoagulable conditions. The production of platelet-derived EVs can be stimulated by several pathological reactions, such as those due to dengue virus [31] or lipopolysaccharide [32]. COVID-19 might have similar stimulatory effects to promote platelet-derived EV level elevation and induce a hypercoagulable status. The levels of endothelium- and platelet-derived EVs in patients with antiphospholipid syndrome, another type of hypercoagulable disease, were higher than those in healthy people [33], which is a trend similar to that observed in the present study.

However, the CD62P^+^ EV levels, another type of platelet-derived EV, did not differ in any of the comparisons. We assume two possible explanations. First, P-selectin is a specific marker for platelet activation [34]. However, P-selectin is also expressed in activated endothelial cells, forming endothelial–leukocyte interactions [35]. This might affect the finding that CD62P-positive EVs among two or three groups were not statistically different. Second, these results might suggest that CD41^+^ EVs are derived from megakaryocytes [29]. In patients with COVID-19, the number of megakaryocytes increases, and their function becomes aberrant [36]. Another study clarified that among SARS-CoV-2-infected patients’ megakaryocytes, the infected megakaryocytes were detected more frequently in the blood and bronchoalveolar lavage samples of COVID-19 non-survivors [37]. In this previous study, the infected megakaryocytes were also detected more frequently in the bronchoalveolar lavage samples from COVID-19 non-survivors.

Based on these insights, the elevation in CD41^+^ EV levels may reflect an increase in the production of lung-migrated megakaryocytes. The lung is increasingly recognized as an important organ for platelet production by the migrated megakaryocytes [38]. Moreover, megakaryocytes secrete several growth factors and cytokines, which might cause severe inflammation [39]. Accordingly, the CD41^+^ EV level elevation may indicate increased production of lung-migrated megakaryocytes, which might be useful for evaluating megakaryocyte dysfunction in COVID-19 patients. Additionally, platelet- or megakaryocyte-derived EVs are associated with the delivery of co-receptors of the human immunodeficiency virus (HIV) and the development of HIV infection [40]. HIV is an RNA virus, and these EVs might be associated with the development of COVID-19 infection, which is acquired due to SARS-CoV-2, which is also an RNA virus.

The coagulopathy group did not show higher EV levels than the non-coagulopathy group. This result may be a consequence of the small sample size. The effect size may be smaller than expected, and a slight difference in EVs may affect the development of coagulopathy. Additionally, several studies have reported elevated CD62E^+^ EV and TF^+^ EV levels [41,42]; however, this study did not observe an elevation in the levels of these types of EVs. This difference might be due to the disease severity of our cohort, as 20% of the patients required advanced respiratory support. Furthermore, the low mortality rate due to COVID-19 in Japan and racial differences might affect the low EV levels [43,44]. Therefore, a large observational study is needed to determine the differences in EV levels between the COVID-19 coagulopathy and COVID-19 non-coagulopathy patients.

This study has some limitations. First, many patients were transferred from other hospitals for intensive care, such as mechanical ventilation and extracorporeal membrane oxygenation, and had a longer COVID-19 duration. Patients in the coagulopathy group might have been transferred more frequently than those in the non-coagulopathy group. In this case, the duration from the onset of COVID-19 to the timing of blood sample collection might have differed between the two groups. Second, our experimental methodology included additional wash and EV concentration processes for the inactivation of COVID-19, which possibly affected the EV counts. Therefore, we used BD LSRFortessa for the analysis of EVs. When measurements are conducted using flow cytometry, the accuracy is reduced for sizes below 600 nm [45]. This may affect our research methodology. Third, our study did not further examine the mechanisms underlying the COVID-19 infection of the megakaryocytes. Therefore, the origin of CD41+ EV levels remains unclear. In our study, we did not examine the expression of full-length filamin A [29] or explore megakaryocyte existence in patients’ lungs. Fourth, our study only provides information on the association between COVID-19 and increased CD41+ EV levels, and provides no evidence of a causal relationship between increased EV levels and the development of COVID-19 coagulopathy. Finally, we did not measure the von Willebrand factor and Lupus anticoagulant levels to meticulously evaluate coagulopathy. Hence, future studies should examine the causal relationship by meticulously measuring the EV levels and clarifying the origin of CD41+ EVs. Regardless of these limitations, our research results provide useful information regarding the levels of EV in COVID-19 patients with coagulopathy.

## 5. Conclusions

In conclusion, no difference was found in the EV levels between the COVID-19 coagulopathy and COVID-19 non-coagulopathy groups. However, among the three study groups, the CD41^+^ EV levels were significantly higher in the COVID-19 group than in the healthy volunteer group. Large prospective studies are needed to elucidate the mechanism underlying the association of COVID-19 with higher CD41^+^ EV levels.

## Figures and Tables

**Table 1:** Baseline characteristics of the COVID-19 coagulopathy group and COVID-19 non-coagulopathy group.

	Non-Coagulopathy Group n = 51	Coagulopathy Group n = 48	
Variables			*p*-Value
Male, n (%)	35 (68.6)	33 (68.8)	1
Age, years, mean (SD)	62 (17)	71 (12)	0.008
Height, cm, median [IQR]	167.0 [155.3–171.9]	164.50 [158.25–170.25]	0.566
Body weight, kg, median [IQR]	66.0 [57.25–80.60]	65.40 [56.33–75.48]	0.583
Body mass index, kg/m^2^ [IQR]	24.5 (21.5–27.9)	23.6 (21.0–27.8)	0.856
Current smoker, n (%)	27 (56.2)	27 (58.7)	0.837
Comorbidities at admission			
Heart failure, n (%)	3 (5.9)	2 (4.2)	1
Brain vessel disease, n (%)	4 (7.8)	7 (14.6)	0.348
Dementia, n (%)	2 (3.9)	9 (18.8)	0.025
Chronic obstructive pulmonary disease, n (%)	6 (11.8)	6 (12.5)	1
Tissue disease, n (%)	2 (3.9)	2 (4.2)	1
Gastric ulcer, n (%)	1 (2.0)	3 (6.2)	0.352
Diabetes mellitus without complication, n (%)	12 (23.5)	11 (22.9)	1
Diabetes mellitus with complication, n (%)	1 (2.0)	2 (4.2)	0.61
Chronic kidney disease, n (%)	1 (2.0)	5 (10.4)	0.105
Dialysis, n (%)	0 (0.0)	3 (6.2)	0.11
Venous thromboembolism (%)	1 (2.0)	0 (0.0)	1
Charlson Comorbidity Index, median [IQR]	1.0 [0.0–1.0]	1.0 [0.0–2.0]	0.046
Medication before admission			
Antiplatelet therapy, n (%)	1 (2.0)	5 (10.4)	0.105
Anticoagulant therapy, n (%)	5 (9.8)	1 (2.1)	0.206
Blood examination data			
White blood cell, ×10^3^ counts/µL, median [IQR]	5.2 [3.80–6.50]	6.10 [4.759.00]	0.04
Platelet, ×10^3^ counts/µL, median [IQR]	170.0 [148.0–237.0]	188.0 [129.25–267.25]	0.894
Lactate dehydrogenase, IU/L, median [IQR]	237 [201–292]	311 [253–419]	<0.001
Total bilirubin, mg/dL, median [IQR]	0.7 [0.5–0.8]	0.6 [0.4–0.7]	0.251
Total protein, g/dL, median [IQR]	6.80 [6.35–7.20]	6.50 [6.00–6.97]	0.035
Albumin, g/dL, mean (SD)	3.73 (0.57)	3.11 (0.60)	<0.001
Blood urea nitrogen, mg/dL, median [IQR]	15.00 [11.00–19.50]	21.50 [15.75–28.25]	<0.001
Creatinine, mg/dL, median [IQR]	0.85 [0.65–0.98]	0.96 [0.74–1.10]	0.083
C-reactive protein, mg/dL, median [IQR]	3.27 [0.99–9.12]	5.89 [3.44–10.97]	0.012
APTT, second, median [IQR]	30.2 [26.8–34.4]	30.8 [28.0–35.1]	0.477
Fibrinogen, mg/dL, median [IQR]	514 [411–604]	575 [474–620]	0.036
D-dimer mg/dL, median [IQR]	0.50 [0.50–0.75]	1.90 [1.45–3.52]	<0.001
Clinical progression at admission (%)			<0.001
Hospitalized, no oxygen therapy	37 (72.5)	12 (25.0)	
Hospitalized, oxygen by mask or nasal prongs	10 (19.6)	20 (41.7)	
Mechanical ventilation (PaO_2_/FiO_2_ ratio >150)	1 (2.0)	9 (18.8)	
Mechanical ventilation (PaO_2_/FiO_2_ ratio >150) or vasopressors	3 (5.9)	4 (8.3)	
Mechanical ventilation PaO_2_/FiO_2_ ratio <150 + vasopressor, dialysis, or ECMO	0 (0.0)	3 (6.2)	
Worst clinical progression during admission, n (%)			<0.001
Hospitalized, no oxygen therapy	19 (37.3)	7 (14.6)	
Hospitalized, oxygen by mask or nasal prongs	28 (54.9)	16 (33.3)	
Hospitalized, oxygen by NIV or high flow	0 (0.0)	4 (8.3)	
Mechanical ventilation (PaO_2_/FiO_2_ ratio > 150)	1 (2.0)	12 (25.0)	
Mechanical ventilation (PaO_2_/FiO_2_ ratio > 150) or vasopressors	2 (3.9)	2 (3.9)	
Mechanical ventilation (PaO_2_/FiO_2_ ratio > 150) + vasopressor, dialysis, or ECMO	0 (0.0)	2 (3.9)	
Deceased	1 (2.0)	5 (10.4)	
Length of hospital stay, days, median [IQR]	11 [8–15]	14.5 [10–41.5]	0.004
Length of intensive care unit stay, days, (median [IQR])	0 [0,0]	0 [0–10]	<0.001
Outcome at discharge, n (%)			<0.001
Home discharge	46 (90.2)	25 (52.1)	
Transfer to another hospital	4 (7.8)	18 (37.5)	
Death	1 (2.0)	5 (10.4)	

SD, standard deviation; IQR, interquartile range; APTT, activated partial thromboplastin time; PaO_2_/FiO_2_ ratio, ratio of arterial oxygen partial pressure to fractional inspired oxygen; ECMO, extracorporeal membrane oxygenation; NIV, noninvasive ventilation.

**Table 2:** Comparison of EV levels between 10 healthy volunteers and 10 COVID-19 coagulopathy patients.

	Volunteer Group n = 10	Coagulopathy Group n = 10	
Measured EVs			*p*-Value
CD31⁺/CD41⁻ EVs, counts/µL, median [IQR]	123.32 [62.38–178.71]	162.90 [103.97–223.73]	0.226
CD31⁺/CD41^−^/CD142⁺ EVs, counts/µL, median [IQR]	3.52 [0.00–7.25]	0.00 [0.00–6.90]	0.62
CD41⁺ EVs, counts/µL, median [IQR]	184.33 [150.12–254.06]	1079.85 [957.35–4030.22]	**0.002**
CD41⁺/CD142⁺ EVs, counts/µL, median [IQR]	11.92 [6.31–21.60]	16.25 [8.40–31.15]	0.405
CD62P⁺ EVs, counts/µL, median [IQR]	203.23 [157.48–349.14]	470.60 [184.40–695.50]	0.199
CD62P⁺/CD142⁺ EVs, counts/µL, median [IQR]	24.81 [20.73–58.17]	32.85 [19.83–81.17]	0.571
CD16b⁺ EVs, counts/µL, median [IQR]	45.90 [26.05–141.44]	92.20 [45.65–360.20]	0.199
CD16b⁺/CD142⁺ EVs, counts/µL, median [IQR]	10.94 [0.00–49.18]	12.00 [6.68–32.73]	0.818
CD62E⁺ EVs, counts/µL, median [IQR]	193.36 [116.00–271.04]	327.75 [292.97–641.45]	0.082
CD62E⁺/CD142⁺ EVs, counts/µL, median [IQR]	28.47 [23.11–36.33]	41.10 [27.00–181.72]	0.257
CD14⁺ EVs, counts/µL, median [IQR]	116.61 [71.39–241.40]	268.65 [192.45–643.05]	**0.034**
CD14⁺/CD142⁺ EVs, counts/µL, median [IQR]	35.70 [21.28–42.40]	60.95 [20.70–102.80]	0.384
CD142⁺ EVs, counts/µL, median [IQR]	44.29 [36.18–72.99]	112.72 [63.88–160.96]	**0.008**

EVs, extracellular vesicles; IQR, interquartile range; COVID-19, coronavirus disease 2019. Significant *p*-values (*p*  <  0.05) are shown in bold.

**Table 3:** Comparison of EV levels between the COVID-19 non-coagulopathy and COVID-19 coagulopathy groups.

	Non-Coagulopathy Group n = 51	Coagulopathy Group n = 48	
Measured EVs			*p*-Value
CD31⁺ EVs, counts/µL, median [IQR]	72.60 [28.45–123.80]	62.00 [13.88–103.97]	0.37
CD31⁺/CD142⁺ EVs, counts/µL, median [IQR]	0.00 [0.00–0.00]	0.00 [0.00–0.00]	0.617
CD41⁺ EVs, counts/µL, median [IQR]	419.80 [152.70–949.85]	549.90 [255.05–984.65]	0.433
CD41⁺/CD142⁺ EVs, counts/µL, median [IQR]	6.40 [0.00–21.40]	5.90 [0.00–28.00]	0.833
CD62P⁺ EVs, counts/µL, median [IQR]	252.40 [152.30–346.60]	197.90 [144.05–333.92]	0.519
CD62P⁺/CD142⁺ EVs, counts/µL, median [IQR]	18.40 [7.30–31.20]	16.70 [5.88–35.35]	0.602
CD16b⁺ EVs, counts/µL, median [IQR]	133.20 [64.90–243.30]	115.05 [52.25–274.88]	0.889
CD16b⁺/CD142⁺ EVs, counts/µL, median [IQR]	6.80 [0.00–17.10]	7.35 [5.42–20.08]	0.159
CD62E⁺ EVs, counts/µL, median [IQR]	223.80 [125.60–565.80]	203.35 [77.28–515.45]	0.421
CD62E⁺/CD142⁺ EVs, counts/µL, median [IQR]	73.10 [21.40–147.50]	43.85 [24.02–150.62]	0.609
CD14⁺ EVs, counts/µL, median [IQR]	234.20 [88.35–373.00]	186.35 [110.15–319.28]	0.642
CD14⁺/CD142⁺ EVs, counts/µL, median [IQR]	24.70 [11.95–51.05]	18.40 [5.60–37.52]	0.292
CD142⁺ EVs, counts/µL, median [IQR]	60.15 [27.17–111.66]	61.23 [36.09–103.05]	0.682

EVs, extracellular vesicles; IQR, interquartile range; COVID-19, coronavirus disease 2019.

## Data Availability

Datasets are available on request to the corresponding author.

## Supplementary Materials

The following supporting information can be downloaded at https://www.mdpi.com/article/10.3390/jcm12103460/s1. Figure S1: Staining strategy used for the flow cytometric analysis of CD31^+^/CD41^−^ EVs, CD41^+^ EVs, and CD142^+^ EVs in human plasma. Figure S2: Staining strategy used for the flow cytometric analysis of CD62P^+^ EVs, CD16b^+^ EVs, and CD142^+^ EVs in human plasma. Figure S3: Staining strategy used for the flow cytometric analysis of CD62E^+^ EVs, CD41^+^ EVs, and CD142^+^ EVs in human plasma. Figure S4: Staining strategy used for the flow cytometric analysis of CD14^+^ EVs, CD41^+^ EVs, and CD142^+^ EVs in human plasma.
