# Supplementary material for: Circulating Extracellular Vesicle Levels in Patients with Coronavirus Disease 2019 Coagulopathy: A Prospective Cohort Study
Source: J Clin Med. 2023 May 14;12(10):3460. doi: 10.3390/jcm12103460 (PMC10218835; doi:10.3390/jcm12103460)
Supplement: Supplementary file 1 [file jcm-12-03460-s001.zip › jcm-2350856-supplementary.pdf]

Journal of
Clinical Medicine

MDPI

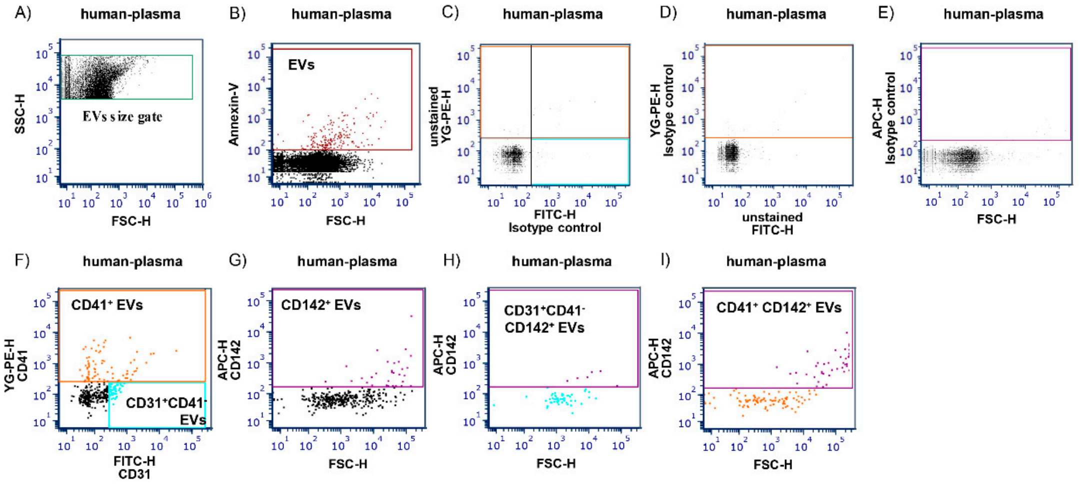

Figure S1. Staining strategy used for the flow cytometric analysis of CD31^{+}/CD41^{-} EVs, CD41^{+} EVs, and CD142^{+} EVs in human plasma. (A) FSC/SSC dot plot of human plasma. The EV size gate was defined as a green-lined rectangle using a mix of fluorescent beads of various diameters (Megamix-Plus SSC™). (B) Representative image of the flow cytometric analysis using Annexin V to identify EVs in human plasma. The EV size gate was set as a brown-lined rectangle. (C–G) Staining strategy for detecting CD31+/CD41^{-} EVs, CD41+ EVs, and CD142+ EVs in human plasma. The black dots represent EVs. (C) Dot plot of staining with FITC-conjugated isotype control antibody. (D) Dot plot of staining with PE-conjugated isotype control antibody. (E) Dot plot of staining with APC-conjugated isotype control antibody. (F) Dot plot of staining with FITC-conjugated anti-human CD31 antibody and PE-conjugated anti-human CD41 antibody. CD41+ EVs were identified as orange dots within an orange-lined rectangle. CD31+/D41^{-} EVs were identified as sky-blue dots within a sky blue-lined rectangle. (G) Dot plot of staining with the APC-conjugated anti-human CD142 antibody. CD142+ EVs were identified as purple dots within a purple-lined rectangle. (H) Dot plot of CD31+/CD41- EVs (sky blue dots) staining with APC-conjugated anti-human CD142 antibody. CD31+/CD41-/CD142+ EVs were identified as purple dots within the purple-lined square. (I) Dot plot of CD41+ EVs (orange dots) staining with APC-conjugated anti-human CD142 antibody. CD41+/CD142+ EVs were identified as purple dots within the purple-lined square.EVs, extracellular vesicles; FSC, forward scatter; SSC, side scatter; FITC, fluorescein isothiocyanate; PE, phycoerythrin; APC, allophycocyanin

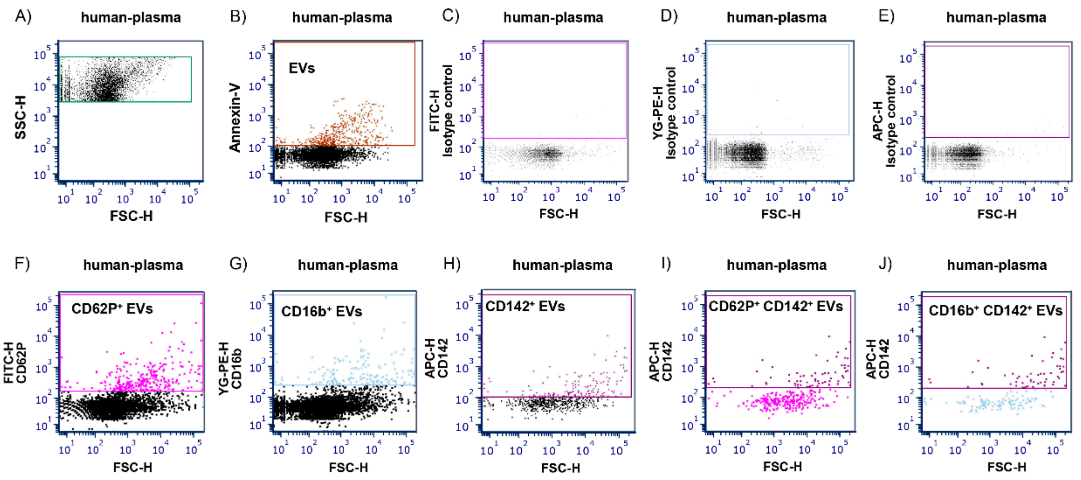

Figure S2. Staining strategy used for the flow cytometric analysis of CD62P⁺ EVs, CD16b⁺ EVs, and CD142⁺ EVs in human plasma. (A) FSC/SSC dot plot of human plasma. The EV size gate was set as a green-lined rectangle using a mix of fluorescent beads of various diameters (Megamix-Plus SSC™). (B) Representative image of flow cytometric analysis using Annexin V to identify EVs in human plasma. The EV size gate was set as a brown-lined rectangle. (C–H) Staining strategy for detecting CD62P+ EVs, CD16b+ EVs, and CD142+ EVs in human plasma. The black dots represent EVs. (C) Dot plot of staining with FITC-conjugated isotype control antibody. (D) Dot plot of staining with PE-conjugated isotype control antibody. (E) Dot plot of staining with APC-conjugated isotype control antibody. (F) Dot plot of staining with FITC-conjugated anti-human CD62P antibody. CD62P+ EVs were identified as pink dots within a pink-lined rectangle. (G) Dot plot of staining with PE-conjugated anti-human CD16b antibody. CD16b+ EVs were identified as light blue dots within a light blue-lined rectangle. (H) A dot plot of staining with the APC-conjugated anti-human CD142 antibody. CD142+ EVs were identified as purple dots within a purple-lined rectangle. (I) Dot plot of CD62P+ EVs (pink dots) staining with APC-conjugated anti-human CD142 antibody. CD62P+/CD142+ EVs were identified as purple dots within the purple-lined square. (J) Dot plot of CD16b+ EVs (light-blue dots) staining with APC-conjugated anti-human CD142 antibody. CD16b+/CD142+ EVs were identified as purple dots within the purple-lined square. EVs, extracellular vesicles; FSC, forward scatter; SSC, side scatter; FITC, fluorescein isothiocyanate; PE, phycoerythrin; APC, allophycocyanin

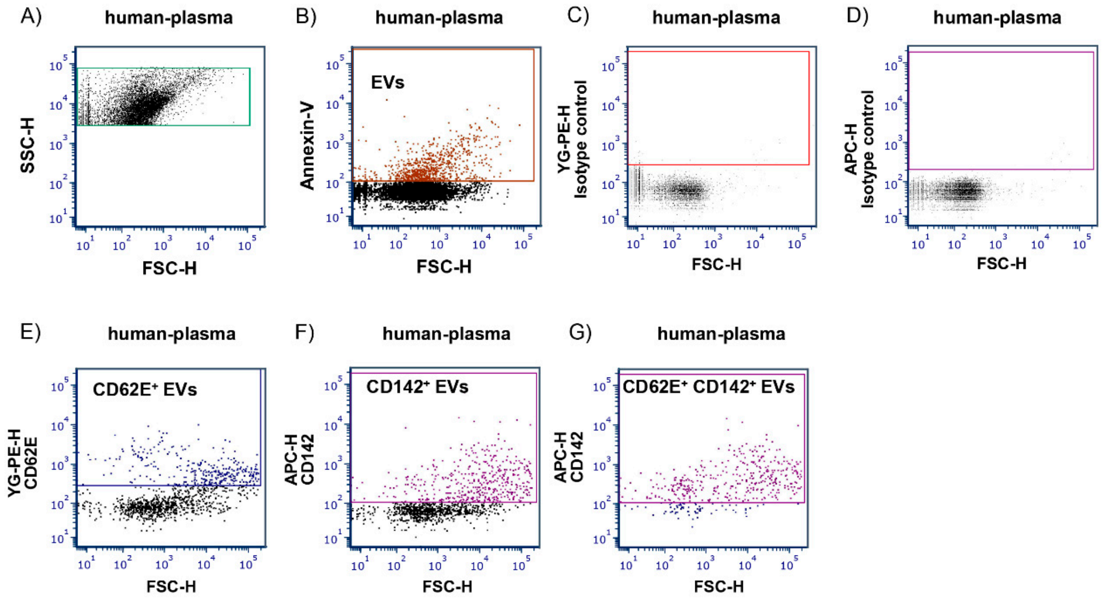

Figure S3. Staining strategy used for the flow cytometric analysis of CD62E⁺ EVs, CD41⁺ EVs, and CD142⁺ EVs in human plasma. (A) FSC/SSC dot plot of human plasma. The EV size gate was set as a green-lined rectangle using a mix of fluorescent beads of various diameters (Megamix-Plus SSC™). (B) Representative image of the flow cytometric analysis using Annexin V to identify EVs in human plasma. The EV size gate was set as a brown-lined rectangle. (C–F) Staining strategy for detecting CD62E+ EVs and CD142+ EVs in human plasma. The black dots represent EVs. (C) Dot plot of staining with PE-conjugated isotype control antibody. (D) Dot plot of staining with APC-conjugated isotype control antibody. (E) Dot plot of staining with PE-conjugated anti-human CD62E antibody. CD62E+ EVs were identified as blue dots within a blue-lined rectangle. (F) Dot plot of staining with the APC-conjugated anti-human CD142 antibody. CD142+ EVs were identified as purple dots within a purple-lined rectangle. (G) Dot plot of CD62E+ EVs (blue dots) staining with APC-conjugated anti-human CD142 antibody. CD62E+/CD142+ EVs were identified as purple dots within the purple-lined square. EVs, extracellular vesicles; FSC, forward scatter; SSC, side scatter; PE, phycoerythrin; APC, allophycocyanin.

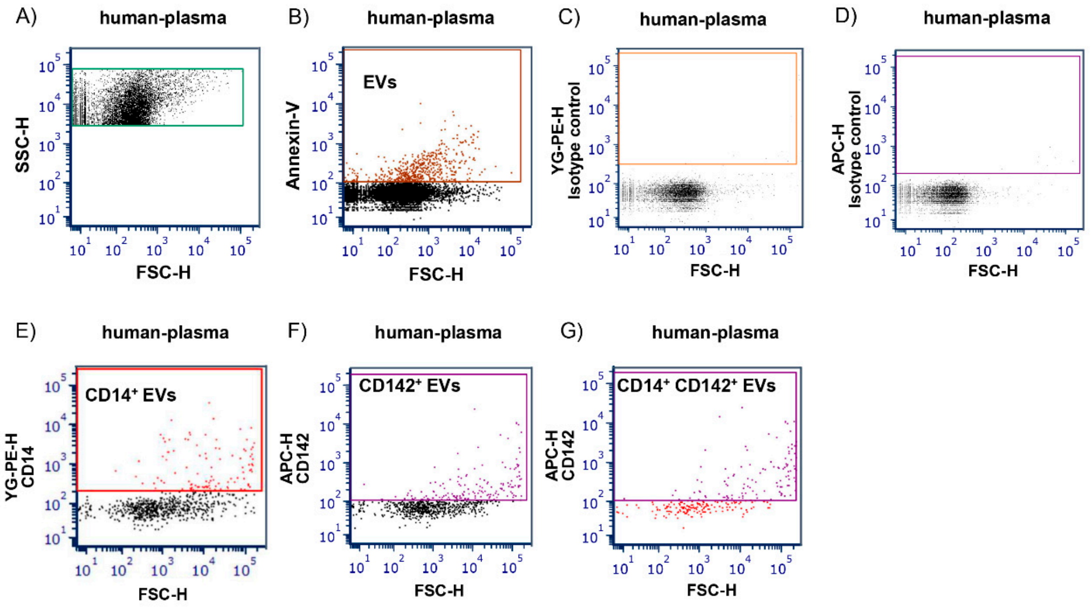

Figure S4. Staining strategy used for the flow cytometric analysis of CD14^{+} EVs, CD41^{+} EVs, and CD142^{+} EVs in human plasma. (A) FSC/SSC dot plot of human plasma. The EV size gate was set as a green-lined rectangle using a mix of fluorescent beads of various diameters (Megamix-Plus SSC™). (B) Representative image of flow cytometric analysis using Annexin V to identify EVs in human plasma. The EV size gate was set as a brown-lined rectangle. (C–F) Staining strategy for detecting CD14+ EVs and CD142+ EVs in human plasma. The black dots represent EVs. (C) Dot plot of staining with PE-conjugated isotype control antibody. (D) Dot plot of staining with APC-conjugated isotype control antibody. (E) Dot plot of staining with PE-conjugated anti-human CD14 antibody. CD62E+ EVs were identified as red dots within a red-lined rectangle. (F) Dot plot of staining with the APC-conjugated anti-human CD142 antibody. CD142+ EVs were identified as purple dots within a purple-lined rectangle. (G) Dot plot of CD14+ EVs (red dots) staining with APC-conjugated anti-human CD142 antibody. CD14+/CD142+ EVs were identified as purple dots within the purple-lined square. EVs, extracellular vesicles; FSC, forward scatter; SSC, side scatter; PE, phycoerythrin; APC, allophycocyanin.
